# *catena*-Poly[[[aqua­pyridine­zinc(II)]-μ_2_-3,3′-(*p*-phenyl­ene)diacrylato] pyridine solvate]

**DOI:** 10.1107/S1600536810033167

**Published:** 2010-08-28

**Authors:** Dongpo Su, Desheng Song, Zhiyong Fu

**Affiliations:** a School of Chemistry and Chemical Engineering, South China University of Technology, Guangzhou, People’s Republic of China

## Abstract

The title compound, {[Zn(C_12_H_8_O_4_)(C_5_H_5_N)(H_2_O)]·C_5_H_5_N}_*n*_, has been prepared by hydro­thermal reaction. The Zn^II^ atom is six-coordinated by four carboxyl­ate O atoms of two *p*-phenylenediacrylate (ppda^2−^) ligands, one N atom of a pyridine mol­ecule and one O atom of a water mol­ecule in a distorted octa­hedral environment. The carboxyl­ate groups of the ppda^2−^ anions are in a bridging–chelating mode, in which two O atoms chelate one Zn^2+^ ion. These connections result in an extended chain structure. Parallel packing of the chains forms a two-dimensional network with inter­molecular edge-to-face inter­actions. Further linkages between the layers through O—H⋯O hydrogen-bonding inter­actions result in a three-dimensional supra­molecular architecture with one-dimensional recta­nglar channels.

## Related literature

For the applications of metal-organic frameworks, see: Li *et al.* (2009 ▶); Zhang *et al.* (2010 ▶). For the rational design and synthesis of coordination polymers by covalent interactions or supra­molecular contacts, see: Jose *et al.* (2010 ▶); Zeng *et al.* (2010 ▶). For a similar complex, see: Sun *et al.* (2009 ▶).
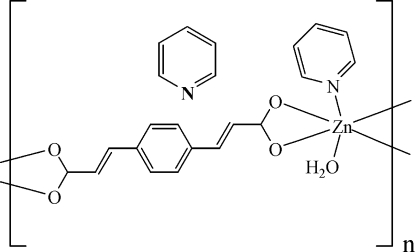


## Experimental

### 

#### Crystal data

[Zn(C_12_H_8_O_4_)(C_5_H_5_N)(H_2_O)]·C_5_H_5_N
*M* *_r_* = 457.77
Monoclinic, 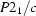

*a* = 10.2132 (15) Å
*b* = 17.375 (3) Å
*c* = 12.8144 (19) Å
β = 112.360 (2)°
*V* = 2103.0 (5) Å^3^
*Z* = 4
Mo *K*α radiation
μ = 1.20 mm^−1^
*T* = 110 K
0.30 × 0.16 × 0.15 mm

#### Data collection

Bruker SMART CCD diffractometer
Absorption correction: multi-scan (*SADABS*; Sheldrick, 1996 ▶) *T*_min_ = 0.74, *T*_max_ = 0.85
9012 measured reflections
3657 independent reflections
2923 reflections with *I* > 2σ(*I*)
*R*_int_ = 0.026

#### Refinement

*R*[*F* ^2^ > 2σ(*F* ^2^)] = 0.035
*wR*(*F* ^2^) = 0.088
*S* = 1.05
3657 reflections
267 parameters
1 restraint
H atoms treated by a mixture of independent and constrained refinement
Δρ_max_ = 0.91 e Å^−3^
Δρ_min_ = −0.81 e Å^−3^

### 

Data collection: *SMART* (Bruker, 1998 ▶); cell refinement: *SAINT* (Bruker, 1998 ▶); data reduction: *SAINT*; program(s) used to solve structure: *SHELXS97* (Sheldrick, 2008 ▶); program(s) used to refine structure: *SHELXL97* (Sheldrick, 2008 ▶); molecular graphics: *SHELXTL* (Sheldrick, 2008 ▶); software used to prepare material for publication: *SHELXTL*.

## Supplementary Material

Crystal structure: contains datablocks global, I. DOI: 10.1107/S1600536810033167/pb2038sup1.cif

Structure factors: contains datablocks I. DOI: 10.1107/S1600536810033167/pb2038Isup2.hkl

Additional supplementary materials:  crystallographic information; 3D view; checkCIF report

## Figures and Tables

**Table 1:** Selected geometric parameters (Å, °)

Zn1—N1	2.093 (2)
Zn1—O5	2.0288 (19)
Zn1—O4	2.0324 (18)
Zn1—O2^i^	2.0368 (18)
Zn1—O1^i^	2.3019 (18)
Zn1—O3	2.4099 (19)

Symmetry code: (i) 
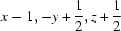
.

**Table 2:** Hydrogen-bond geometry (Å, °)

*D*—H⋯*A*	*D*—H	H⋯*A*	*D*⋯*A*	*D*—H⋯*A*
O5—H1⋯O4^ii^	0.80 (4)	1.94 (4)	2.743 (4)	172 (4)

Symmetry code: (ii) 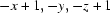.

**Fig. 1.**
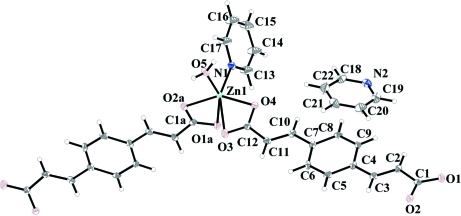
The molecular structure of (I), with atom labels and 50% probability displacement ellipsoids for non-H atoms.

**Fig. 2.**
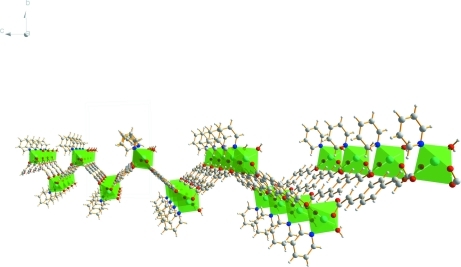
View of the two-dimensional supramolecular layer of the title compound.

**Fig. 3.**
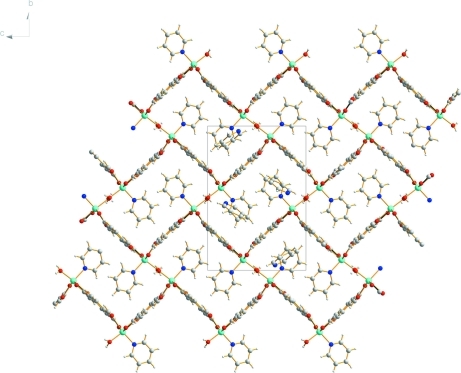
Three-dimensional supramolecular network of the title complex.

## supplementary crystallographic information

### Comment

Metal-organic frameworks (MOFs) became one of the most active research areas in chemistry and materials in recent years due to their intriguing variety of architectures as well as promising applications as functional materials (Li *et al.*, 2009; Zhang *et al.*, 2010). One of the current interesting topics is to rationally design and synthesize coordination polymers and supramolecular organization by coordinated covalent bonds or supramolecular contacts (Zeng *et al.*, 2010; Jose *et al.*, 2010). Herein, we report the synthesis and characterization of a new metal organic framework with three-dimensional supramolecular structural motif. In the title compound, the Zn^II^ center is six-coordinated in a distorted octahedral geometry (Figure 1), surrounded by O1, O2, O4 from two ppda^2-^ ligand and O5 from a water molecule in the equatorial plane, and N1, O3 of pyridine molecule and ppda^2-^ ligand respectively in the axial position. The ppda^2-^ anion adopts a bridging coordination mode, interconnect with the zinc ions forming a 1-D infinite chain. The shortest distance between the neighbour zinc centers is 15.26 Å. The parallel chains are arranged into a two-dimensional network by intermolecular edge-to-face C—H···pi interactions (Figure 2). Two neighboring pyridine molecules from adjacent chains are parallel and form a dihedral angle of 58.1° with the plane of uncoordinated pyridine molecule. C—H···pi interactions exist between uncoordinated pyridine molecule and coordinated pyridine molecules. The C19—H19A and C22—H22A groups point to the center of adjacent pyridine rings, with H···centroid distances 2.9408 (3) and 3.3096 (5) Å. These two-dimensional networks are further linked *via* interlayer strong O—H···O hydrogen-bonding interactions, forming a three-dimensional supramolecular architecture with one-dimensional rectangle-shaped channels along the *a* direction (Figure 3). The H1···O4 distance is 1.8628Å and the O5—H1···O4 bond angle is 171.58°. Guest pyridine molecules are situated in the cavities.

### Experimental

A mixture of H_2_ppda (0.0218 g, 0.1 mmol), Zn(OAc)_2_.2H_2_O (0.0219 g, 0.1 mmol), 4,4,-bpy (0.0156 g, 0.1 mmol), and py/H_2_O (1:3, 12 ml) was sealed in a 25 ml Teflon-lined bomb and heated at 353 K for 48 h. The reaction mixture was then allowed to cool to room temperature at a rate of 3 K/h. Colorless block-shape crystals were obtained.

### Refinement

H atoms were positioned geometrically and refined using a riding model, with C—H = 0.93–0.97 Å and with *U*_iso_(H) = 1.2 (1.5 for methyl groups) times *U*_eq_(C). The non-hydrogen atoms were refined anisotropically. 34 low-theta reflections were omitted from the data set. These Low-theta reflections which calculate large but have a near-zero Fobs, might have been obscured by the beamstop. They were omitted for a well refinement. A restraint was applied for the O5 and H2 atom with O5—H2 = 0.82 Å.

### Crystal data

**Table:**

[Zn(C_12_H_8_O_4_)(C_5_H_5_N)(H_2_O)]·C_5_H_5_N	*F*(000) = 944
*M**_r_* = 457.77	*D*_x_ = 1.446 Mg m^−^^3^
Monoclinic, *P*2_1_/*c*	Mo *K*α radiation, λ = 0.71073 Å
Hall symbol: -P 2ybc	Cell parameters from 3657 reflections
*a* = 10.2132 (15) Å	θ = 2.9–25.0°
*b* = 17.375 (3) Å	µ = 1.20 mm^−^^1^
*c* = 12.8144 (19) Å	*T* = 110 K
β = 112.360 (2)°	Block, colorless
*V* = 2103.0 (5) Å^3^	0.30 × 0.16 × 0.15 mm
*Z* = 4	

### Data collection

**Table:**

Bruker SMART CCD diffractometer	3657 independent reflections
Radiation source: fine-focus sealed tube	2923 reflections with *I* > 2σ(*I*)
graphite	*R*_int_ = 0.026
ω scans	θ_max_ = 25.0°, θ_min_ = 2.9°
Absorption correction: multi-scan (*SADABS*; Sheldrick, 1996)	*h* = −12→12
*T*_min_ = 0.74, *T*_max_ = 0.85	*k* = −20→15
9012 measured reflections	*l* = −11→15

### Refinement

**Table:**

Refinement on *F*^2^	Primary atom site location: structure-invariant direct methods
Least-squares matrix: full	Secondary atom site location: difference Fourier map
*R*[*F*^2^ > 2σ(*F*^2^)] = 0.035	Hydrogen site location: inferred from neighbouring sites
*wR*(*F*^2^) = 0.088	H atoms treated by a mixture of independent and constrained refinement
*S* = 1.05	*w* = 1/[σ^2^(*F*_o_^2^) + (0.044*P*)^2^ + 1.4847*P*] where *P* = (*F*_o_^2^ + 2*F*_c_^2^)/3
3657 reflections	(Δ/σ)_max_ < 0.001
267 parameters	Δρ_max_ = 0.91 e Å^−^^3^
1 restraint	Δρ_min_ = −0.81 e Å^−^^3^

### Special details

**Table:**

Geometry. All e.s.d.'s (except the e.s.d. in the dihedral angle between two l.s. planes) are estimated using the full covariance matrix. The cell e.s.d.'s are taken into account individually in the estimation of e.s.d.'s in distances, angles and torsion angles; correlations between e.s.d.'s in cell parameters are only used when they are defined by crystal symmetry. An approximate (isotropic) treatment of cell e.s.d.'s is used for estimating e.s.d.'s involving l.s. planes.
Refinement. Refinement of *F*^2^ against ALL reflections. The weighted *R*-factor *wR* and goodness of fit *S* are based on *F*^2^, conventional *R*-factors *R* are based on *F*, with *F* set to zero for negative *F*^2^. The threshold expression of *F*^2^ > σ(*F*^2^) is used only for calculating *R*-factors(gt) *etc*. and is not relevant to the choice of reflections for refinement. *R*-factors based on *F*^2^ are statistically about twice as large as those based on *F*, and *R*- factors based on ALL data will be even larger.

### Fractional atomic coordinates and isotropic or equivalent isotropic displacement parameters (Å^2^)

**Table:**

	*x*	*y*	*z*	*U*_iso_*/*U*_eq_	
N1	0.5178 (2)	−0.01394 (13)	0.75537 (18)	0.0163 (5)	
C18	0.9544 (3)	0.03667 (18)	0.3132 (2)	0.0260 (7)	
H18A	0.9348	0.0054	0.3663	0.031*	
Zn1	0.41999 (3)	0.072906 (17)	0.63842 (2)	0.01285 (11)	
C1	1.3097 (3)	0.35091 (15)	0.2483 (2)	0.0145 (6)	
O1	1.44021 (18)	0.34052 (11)	0.27971 (15)	0.0177 (4)	
O2	1.24483 (18)	0.39778 (11)	0.16907 (14)	0.0149 (4)	
O3	0.40900 (19)	0.18631 (11)	0.52712 (15)	0.0193 (4)	
O4	0.58751 (18)	0.10624 (10)	0.60053 (14)	0.0148 (4)	
O5	0.3145 (2)	0.00368 (12)	0.50538 (16)	0.0183 (4)	
H2	0.239 (3)	0.0196 (18)	0.451 (2)	0.031 (9)*	
H1	0.351 (4)	−0.026 (2)	0.476 (3)	0.030 (10)*	
C2	1.2295 (3)	0.30907 (16)	0.3060 (2)	0.0155 (6)	
H2A	1.2736	0.2670	0.3539	0.019*	
C3	1.0980 (3)	0.32845 (15)	0.2929 (2)	0.0146 (6)	
H3A	1.0559	0.3692	0.2417	0.018*	
C4	1.0110 (3)	0.29379 (15)	0.3489 (2)	0.0144 (6)	
C5	0.8720 (3)	0.31994 (16)	0.3217 (2)	0.0167 (6)	
H5A	0.8369	0.3606	0.2688	0.020*	
C6	0.7854 (3)	0.28786 (16)	0.3702 (2)	0.0158 (6)	
H6A	0.6909	0.3058	0.3489	0.019*	
C7	0.8350 (3)	0.22904 (15)	0.4506 (2)	0.0137 (5)	
C8	0.9737 (3)	0.20268 (15)	0.4779 (2)	0.0137 (6)	
H8A	1.0089	0.1623	0.5313	0.016*	
C9	1.0604 (3)	0.23431 (15)	0.4286 (2)	0.0141 (5)	
H9A	1.1543	0.2157	0.4489	0.017*	
C10	0.7473 (3)	0.19340 (15)	0.5051 (2)	0.0138 (6)	
H10A	0.7887	0.1517	0.5548	0.017*	
C11	0.6165 (3)	0.21278 (15)	0.4930 (2)	0.0152 (6)	
H11A	0.5755	0.2576	0.4507	0.018*	
C12	0.5322 (3)	0.16709 (15)	0.5429 (2)	0.0141 (6)	
C13	0.6192 (3)	0.00035 (18)	0.8551 (2)	0.0309 (5)	
H13A	0.6482	0.0522	0.8739	0.037*	
C14	0.6844 (4)	−0.05630 (18)	0.9326 (3)	0.0360 (6)	
H14A	0.7576	−0.0436	1.0027	0.043*	
C15	0.6423 (4)	−0.13168 (19)	0.9073 (3)	0.0345 (8)	
H15A	0.6827	−0.1715	0.9607	0.041*	
C16	0.5408 (4)	−0.14810 (19)	0.8033 (3)	0.0360 (6)	
H16A	0.5118	−0.1996	0.7817	0.043*	
C17	0.4820 (4)	−0.08740 (17)	0.7307 (3)	0.0309 (5)	
H17A	0.4116	−0.0988	0.6588	0.037*	
N2	1.0893 (3)	0.05277 (14)	0.3312 (2)	0.0227 (6)	
C19	1.1138 (3)	0.09713 (18)	0.2556 (3)	0.0296 (7)	
H19A	1.2093	0.1091	0.2676	0.035*	
C20	1.0086 (4)	0.12641 (19)	0.1615 (3)	0.0344 (8)	
H20A	1.0309	0.1575	0.1095	0.041*	
C21	0.8701 (3)	0.1097 (2)	0.1440 (3)	0.0330 (8)	
H21A	0.7946	0.1296	0.0802	0.040*	
C22	0.8431 (3)	0.0636 (2)	0.2209 (3)	0.0343 (8)	
H22A	0.7485	0.0505	0.2102	0.041*	

### Atomic displacement parameters (Å^2^)

**Table:**

	*U*^11^	*U*^22^	*U*^33^	*U*^12^	*U*^13^	*U*^23^
N1	0.0178 (12)	0.0153 (12)	0.0166 (11)	0.0013 (10)	0.0073 (9)	0.0012 (9)
C18	0.0300 (17)	0.0237 (16)	0.0258 (16)	−0.0048 (13)	0.0123 (14)	0.0010 (13)
Zn1	0.01274 (17)	0.01317 (17)	0.01527 (17)	0.00028 (13)	0.00828 (12)	0.00003 (13)
C1	0.0161 (14)	0.0127 (14)	0.0160 (13)	−0.0010 (11)	0.0075 (11)	−0.0045 (11)
O1	0.0132 (10)	0.0198 (10)	0.0219 (10)	0.0013 (8)	0.0088 (8)	0.0035 (8)
O2	0.0149 (9)	0.0156 (9)	0.0166 (9)	−0.0006 (8)	0.0087 (8)	0.0029 (8)
O3	0.0166 (10)	0.0215 (11)	0.0244 (10)	0.0026 (8)	0.0130 (8)	0.0056 (8)
O4	0.0153 (9)	0.0145 (10)	0.0174 (9)	0.0008 (8)	0.0093 (8)	0.0014 (8)
O5	0.0181 (11)	0.0179 (11)	0.0179 (10)	0.0039 (9)	0.0058 (9)	−0.0049 (9)
C2	0.0159 (14)	0.0166 (14)	0.0161 (13)	0.0002 (11)	0.0084 (11)	0.0021 (11)
C3	0.0156 (13)	0.0154 (14)	0.0128 (13)	−0.0010 (11)	0.0055 (11)	0.0009 (11)
C4	0.0127 (13)	0.0178 (14)	0.0131 (13)	−0.0024 (11)	0.0056 (11)	−0.0018 (11)
C5	0.0175 (14)	0.0161 (14)	0.0157 (13)	−0.0014 (11)	0.0056 (11)	0.0032 (11)
C6	0.0107 (13)	0.0206 (15)	0.0170 (13)	0.0012 (11)	0.0063 (11)	−0.0006 (11)
C7	0.0124 (13)	0.0158 (14)	0.0134 (13)	−0.0030 (11)	0.0053 (10)	−0.0021 (11)
C8	0.0174 (14)	0.0106 (13)	0.0106 (12)	−0.0006 (11)	0.0024 (11)	0.0009 (10)
C9	0.0110 (13)	0.0157 (14)	0.0157 (13)	0.0002 (11)	0.0054 (10)	−0.0028 (11)
C10	0.0155 (13)	0.0143 (14)	0.0104 (12)	−0.0027 (11)	0.0038 (10)	−0.0003 (10)
C11	0.0190 (14)	0.0118 (13)	0.0163 (13)	−0.0007 (11)	0.0082 (11)	0.0016 (11)
C12	0.0141 (14)	0.0162 (14)	0.0138 (13)	−0.0026 (11)	0.0073 (11)	−0.0051 (11)
C13	0.0386 (13)	0.0196 (12)	0.0240 (11)	−0.0022 (10)	0.0002 (10)	−0.0009 (9)
C14	0.0455 (14)	0.0231 (12)	0.0272 (12)	−0.0026 (11)	0.0000 (11)	−0.0008 (10)
C15	0.047 (2)	0.0245 (17)	0.0240 (16)	0.0069 (15)	0.0042 (15)	0.0071 (13)
C16	0.0455 (14)	0.0231 (12)	0.0272 (12)	−0.0026 (11)	0.0000 (11)	−0.0008 (10)
C17	0.0386 (13)	0.0196 (12)	0.0240 (11)	−0.0022 (10)	0.0002 (10)	−0.0009 (9)
N2	0.0251 (14)	0.0194 (13)	0.0230 (13)	0.0037 (10)	0.0084 (11)	−0.0013 (10)
C19	0.0239 (16)	0.0305 (18)	0.0362 (18)	−0.0024 (14)	0.0135 (14)	0.0017 (14)
C20	0.039 (2)	0.0326 (19)	0.0330 (18)	0.0029 (15)	0.0154 (15)	0.0118 (15)
C21	0.0267 (17)	0.0352 (19)	0.0313 (17)	0.0053 (15)	0.0042 (14)	0.0083 (15)
C22	0.0227 (17)	0.039 (2)	0.0382 (19)	−0.0055 (15)	0.0081 (15)	0.0018 (16)

### Geometric parameters (Å, °)

**Table:**

N1—C13	1.327 (4)	C5—H5A	0.9500
N1—C17	1.332 (4)	C6—C7	1.402 (4)
Zn1—N1	2.093 (2)	C6—H6A	0.9500
C18—N2	1.336 (4)	C7—C8	1.401 (4)
C18—C22	1.374 (4)	C7—C10	1.466 (4)
C18—H18A	0.9500	C8—C9	1.382 (4)
Zn1—O5	2.0288 (19)	C8—H8A	0.9500
Zn1—O4	2.0324 (18)	C9—H9A	0.9500
Zn1—O2^i^	2.0368 (18)	C10—C11	1.328 (4)
Zn1—O1^i^	2.3019 (18)	C10—H10A	0.9500
Zn1—O3	2.4099 (19)	C11—C12	1.484 (4)
Zn1—C1^i^	2.492 (3)	C11—H11A	0.9500
Zn1—C12	2.560 (3)	C13—C14	1.377 (4)
C1—O1	1.250 (3)	C13—H13A	0.9500
C1—O2	1.273 (3)	C14—C15	1.378 (4)
C1—C2	1.485 (4)	C14—H14A	0.9500
C1—Zn1^ii^	2.492 (3)	C15—C16	1.371 (4)
O1—Zn1^ii^	2.3019 (18)	C15—H15A	0.9500
O2—Zn1^ii^	2.0368 (18)	C16—C17	1.383 (4)
O3—C12	1.241 (3)	C16—H16A	0.9500
O4—C12	1.291 (3)	C17—H17A	0.9500
O5—H2	0.864 (18)	N2—C19	1.334 (4)
O5—H1	0.80 (4)	C19—C20	1.372 (4)
C2—C3	1.333 (4)	C19—H19A	0.9500
C2—H2A	0.9500	C20—C21	1.377 (5)
C3—C4	1.466 (4)	C20—H20A	0.9500
C3—H3A	0.9500	C21—C22	1.377 (5)
C4—C5	1.403 (4)	C21—H21A	0.9500
C4—C9	1.405 (4)	C22—H22A	0.9500
C5—C6	1.377 (4)		
			
C13—N1—C17	116.8 (2)	C6—C5—C4	121.3 (2)
C13—N1—Zn1	122.7 (2)	C6—C5—H5A	119.3
C17—N1—Zn1	120.53 (19)	C4—C5—H5A	119.3
N2—C18—C22	122.6 (3)	C5—C6—C7	120.8 (2)
N2—C18—H18A	118.7	C5—C6—H6A	119.6
C22—C18—H18A	118.7	C7—C6—H6A	119.6
O5—Zn1—O4	101.20 (8)	C8—C7—C6	118.0 (2)
O5—Zn1—O2^i^	94.92 (8)	C8—C7—C10	119.1 (2)
O4—Zn1—O2^i^	148.85 (8)	C6—C7—C10	122.9 (2)
N1—Zn1—O5	97.50 (9)	C9—C8—C7	121.3 (2)
O4—Zn1—N1	99.25 (8)	C9—C8—H8A	119.4
O2^i^—Zn1—N1	104.90 (8)	C7—C8—H8A	119.4
O5—Zn1—O1^i^	155.29 (8)	C8—C9—C4	120.6 (2)
O4—Zn1—O1^i^	99.78 (7)	C8—C9—H9A	119.7
O1^i^—Zn1—O2^i^	60.49 (7)	C4—C9—H9A	119.7
N1—Zn1—O1^i^	91.86 (8)	C11—C10—C7	127.3 (2)
O5—Zn1—O3	95.57 (7)	C11—C10—H10A	116.3
O3—Zn1—O4	58.64 (7)	C7—C10—H10A	116.3
O2^i^—Zn1—O3	93.61 (7)	C10—C11—C12	122.5 (2)
N1—Zn1—O3	156.24 (8)	C10—C11—H11A	118.8
O1^i^—Zn1—O3	84.33 (7)	C12—C11—H11A	118.8
O5—Zn1—C1^i^	125.46 (8)	O3—C12—O4	120.6 (2)
O4—Zn1—C1^i^	125.88 (8)	O3—C12—C11	120.3 (2)
O2^i^—Zn1—C1^i^	30.61 (8)	O4—C12—C11	119.0 (2)
N1—Zn1—C1^i^	99.99 (8)	O3—C12—Zn1	68.89 (14)
O1^i^—Zn1—C1^i^	29.89 (7)	O4—C12—Zn1	51.76 (12)
O3—Zn1—C1^i^	88.32 (7)	C11—C12—Zn1	170.73 (19)
O5—Zn1—C12	99.68 (8)	N1—C13—C14	123.2 (3)
O4—Zn1—C12	29.91 (8)	N1—C13—H13A	118.4
O2^i^—Zn1—C12	121.19 (8)	C14—C13—H13A	118.4
N1—Zn1—C12	128.66 (9)	C13—C14—C15	119.1 (3)
O1^i^—Zn1—C12	92.05 (7)	C13—C14—H14A	120.4
O3—Zn1—C12	28.72 (7)	C15—C14—H14A	120.4
C1^i^—Zn1—C12	108.13 (8)	C16—C15—C14	118.7 (3)
O1—C1—O2	121.1 (2)	C16—C15—H15A	120.7
O1—C1—C2	119.4 (2)	C14—C15—H15A	120.7
O2—C1—C2	119.4 (2)	C15—C16—C17	118.0 (3)
O1—C1—Zn1^ii^	66.61 (14)	C15—C16—H16A	121.0
O2—C1—Zn1^ii^	54.53 (12)	C17—C16—H16A	121.0
C2—C1—Zn1^ii^	173.96 (19)	N1—C17—C16	124.2 (3)
C1—O1—Zn1^ii^	83.50 (15)	N1—C17—H17A	117.9
C1—O2—Zn1^ii^	94.86 (15)	C16—C17—H17A	117.9
C12—O3—Zn1	82.39 (15)	C19—N2—C18	117.5 (3)
C12—O4—Zn1	98.33 (15)	N2—C19—C20	123.5 (3)
Zn1—O5—H2	121 (2)	N2—C19—H19A	118.3
Zn1—O5—H1	125 (2)	C20—C19—H19A	118.3
H2—O5—H1	105 (3)	C19—C20—C21	118.5 (3)
C3—C2—C1	122.3 (3)	C19—C20—H20A	120.7
C3—C2—H2A	118.8	C21—C20—H20A	120.7
C1—C2—H2A	118.8	C20—C21—C22	118.7 (3)
C2—C3—C4	127.2 (3)	C20—C21—H21A	120.7
C2—C3—H3A	116.4	C22—C21—H21A	120.7
C4—C3—H3A	116.4	C21—C22—C18	119.2 (3)
C5—C4—C9	117.9 (2)	C21—C22—H22A	120.4
C5—C4—C3	119.3 (2)	C18—C22—H22A	120.4
C9—C4—C3	122.8 (2)		

Symmetry codes: (i) *x*−1, −*y*+1/2, *z*+1/2; (ii) *x*+1, −*y*+1/2, *z*−1/2.

### Hydrogen-bond geometry (Å, °)

**Table:**

*D*—H···*A*	*D*—H	H···*A*	*D*···*A*	*D*—H···*A*
O5—H1···O4^iii^	0.80 (4)	1.94 (4)	2.743 (4)	172 (4)

Symmetry codes: (iii) −*x*+1, −*y*, −*z*+1.
